# A Systematic Literature Review and Bucher Indirect Comparison: Tildrakizumab versus Guselkumab

**DOI:** 10.36469/jheor.2020.13671

**Published:** 2020-07-24

**Authors:** Kristian Garn Du Jardin, Pepi Hurtado Lopez, Mette Lange, Rachael McCool, Silvia Maeso Naval, Sandra Quickert

**Affiliations:** 1 Almirall Nordic, Copenhagen, Denmark; 2 Almirall S.A., Barcelona, Spain; 3 York Health Economics Consortium Ltd, York, United Kingdom; 4 Quantics, Edinburgh, United Kingdom

**Keywords:** systematic literature review, tildrakizumab, guselkumab, risk ratio, meta-analysis, Bucher indirect comparison

## Abstract

**Background:**

Psoriasis is a chronic inflammatory skin condition that impacts quality of life and requires long-term treatment and effective symptom management. Interleukin-23 (IL-23) has emerged as a key player in the pathogenesis of psoriasis and tildrakizumab and guselkumab are both immunomodulatory agents that inhibit the p19 subunit of IL-23. In its pivotal Phase III trial, tildrakizumab demonstrated greater efficacy than etanercept in moderate-to-severe psoriasis. However, there are no head-to-head trials comparing tildrakizumab with guselkumab.

**Methods:**

We conducted a systematic literature review and Bucher indirect comparison of tildrakizumab and guselkumab, using placebo as a common comparator. We searched MEDLINE, MEDLINE In-Process, MEDLINE(R) Daily Epub Ahead of Print, and Cochrane Central Register of Controlled Trials for Phase III randomized controlled trials between 1946 and November 2018. Inclusion criteria were adult patients ≥18 years with moderate-to-severe chronic plaque psoriasis, and intervention with tildrakizumab or guselkumab compared to placebo or best supportive care. Outcomes included were severity of psoriasis as defined by the Psoriasis Area and Severity Index (PASI) 75 and PASI 90, frequency of serious adverse events (SAEs), and treatment discontinuations. Outcomes were evaluated at Weeks 12 to 16 and 24 to 28. Analysis was based on the intent-to-treat population and, for all outcomes, the number of events reported were analyzed as a proportion of the number of patients randomized to ensure consistency across trials.

**Results:**

Overall, 154 unique records were identified. Five studies met the eligibility criteria and were included in the analysis; two tildrakizumab trials (reSURFACE 1 and reSURFACE 2) and three guselkumab trials (VOYAGE 1, VOYAGE 2, and a Japanese study). There was no statistically significant difference between guselkumab and tildrakizumab for PASI 75, PASI 90, SAEs, and rate of discontinuations at either timepoint.

**Conclusion:**

This study assessed the comparative efficacy of tildrakizumab and guselkumab for the treatment of moderate-to-severe psoriasis. Limitations included the limited number of publications, imputation of placebo arm values for Weeks 24 to 28, and limited relevance of the Japanese study. This indirect comparison does not provide evidence that one treatment is superior to the other.

## INTRODUCTION

Psoriasis is a chronic inflammatory skin condition accompanied by considerable quality of life impairment and requires long-term treatment and effective symptom management.^1^–^5^ The global prevalence of psoriasis is estimated to be around 2%–3%^6^ and around one-quarter of people with psoriasis have a moderate-to-severe form of the disease.^7^ Treatment of moderate-to-severe psoriasis requires the use of systemic nonbiological or biological agents, while mild cases may be treatable with topical therapies or phototherapy.^8^,^9^

Interleukin-23 (IL-23) is a key player in the pathogenesis of psoriasis and has been shown to be crucial for the activation and persistence of T-helper 17 (Th17) inflammatory pathways that underpin the disease.^10^ IL-23 is a heterodimeric cytokine composed of a unique p19 subunit and a p40 subunit that is shared with interleukin-12 (IL-12);^11^ genetic loci of IL-23p19 and IL-12/23p40 are known to be associated with psoriasis.^10^ Agents developed to block the IL-12/23p40 subunit have demonstrated efficacy in patients with moderate-to-severe psoriasis.^10^ Subsequent research however demonstrated that IL-23 is the predominant driver of psoriasis pathogenesis, not IL-12; intradermal injection of IL-23 in mouse skin models resulted in development of psoriatic plaque lesions while IL-12 injection did not.^10^ The promoted role of IL-23p19 in psoriasis has since driven the development of agents to selectively target the p19 subunit only. Moreover, preserving IL-12-mediated inflammatory responses may improve safety by modulating only the most relevant immune pathways.^10^

Therapeutic strategies for treatment of psoriasis that focus on selective inhibition of IL-23^12^ have demonstrated efficacy in Phase II and Phase III studies.^13^–^17^ Tildrakizumab is a biological agent developed to selectively target the p19 subunit of IL-23 and is approved for the treatment of moderate-to-severe psoriasis in both the European Union (EU; Ilumetri^®^) and United States (US; Ilumya^®^).^18^ In its pivotal Phase III reSURFACE 1 and reSURFACE 2 trials, the efficacy and safety of tildrakizumab was investigated compared with placebo and etanercept, a tumor necrosis factor inhibitor. Tildrakizumab demonstrated greater efficacy compared with placebo and etanercept and was well tolerated in patients with moderate-to-severe plaque psoriasis.^13^ Results of the reSURFACE 2 trial also demonstrated that tildrakizumab achieved greater efficacy compared with etanercept at Week 28 versus Week 12.^13^ Furthermore, longer-term data from this trial have indicated sustained efficacy up to 148 weeks.^14^,^19^ Exposure-adjusted adverse event incidence rates per 100 subject years were lower in tildrakizumab arms compared with etanercept arms but comparable to placebo.^20^ Since 2019, two other IL-23p19 inhibitors have been approved in the EU and the US for the treatment of moderate-to-severe psoriasis: guselkumab (Tremfya^®^) and risankizumab (Skyrizi^®^).^21^–^24^

This study aimed to address the comparative effectiveness and safety of the IL-23p19 inhibitors tildrakizumab and guselkumab. Guselkumab was selected as a comparator as it was the only other IL-23p19 inhibitor approved at the time of study conception and no published studies comparing the two treatments directly were in existence.^10^,^18^ We report results of an indirect comparison to compare tildrakizumab 100 mg (dosed per approved label on Weeks 0, 4, and every 12 weeks thereafter) and guselkumab 100 mg, using placebo as a common comparator.

## METHODS

### Identification of Relevant Trials

A literature search was conducted to identify trials of tildrakizumab and guselkumab in patients with moderate-to-severe psoriasis. Databases searched were Ovid SP MEDLINE, MEDLINE In-Process, MEDLINE(R) Daily Epub Ahead of Print (date range, 1946 to November 20, 2018), and the Cochrane Central Register of Controlled Trials (CENTRAL; issue 11 of 12, November 2018). Details of the search terms used are included in the Supplementary Material (Table S1). Records were deduplicated using several algorithms.

Search results were screened for relevance based on pre-defined inclusion and exclusion criteria (Table 1). Screening was conducted in two phases: title and abstract review, followed by full text review. Outcomes assessed were: severity of psoriasis as defined by the Psoriasis Area and Severity Index (PASI) with PASI 75 and PASI 90 representing a ≥75% and ≥90% reduction in PASI, respectively; frequency of serious adverse events (SAEs); and treatment discontinuations for any cause. Two reviewers assessed the titles and abstracts independently for relevance to the eligibility criteria and a third adjudicated where there was no consensus. Where results for one study were reported in more than one paper, all related papers were identified and grouped together to ensure that participants in individual studies were only included once. Data were extracted from the primary publication for each study where possible. Data extraction was undertaken by two researchers independently and adjudicated by a third, and risk of bias assessment was conducted using the Cochrane Risk of Bias tool.

### Indirect Comparison Methods

A Bucher indirect comparison^25^ was conducted using placebo as a common comparator in three steps: pairwise meta-analysis of placebo and tildrakizumab to estimate a risk ratio, pairwise meta-analysis of placebo and guselkumab to estimate a risk ratio, and a combination of these risk ratios to estimate a risk ratio of tildrakizumab and guselkumab. A risk ratio >1 indicated that more events were reported for guselkumab. The analysis population of interest was the intent-to-treat (ITT) population. For all outcomes, the number of events reported were analyzed as a proportion of the number of patients randomized to ensure consistency across trials. Analyses were performed using R version 3.5.1. Pairwise meta-analyses were performed using either a random effects (RE) model (where risk ratios from different studies can vary) or using a fixed effect (FE) model (where risk ratios from different studies are modeled without variation). The choice of the model depends on the amount of heterogeneity. For completeness, we report results from both FE and RE models. If a comparison was reported by one study only, then no meta-analysis was undertaken, and the risk ratio reported by the single study was used.

Heterogeneity was quantified using the I^2^ statistic. I^2^ values were calculated to assess the variability in intervention effects among studies and were calculated for each pairwise comparison. I^2^ values between 30% and 60% have been identified as representing moderate heterogeneity.^26^ Absolute risk reductions (ARRs) were calculated based on observed results of guselkumab arms and the mean of the relative risk ratios as calculated earlier. A positive ARR indicated fewer events for patients treated with tildrakizumab.

Outcomes were evaluated at Weeks 12 to 16 and Weeks 24 to 28. Studies discontinued placebo arms at Weeks 12 to 16, and the placebo data from Weeks 12 to 16 were used for imputing values for Weeks 24 to 28. The analyses were conducted with the assumption that it is appropriate to aggregate study results reported at 12 to 16 weeks and at 24 to 28 weeks but with relatively small differences in time of data collection.

## RESULTS

### Literature Search

**Figure 1:**
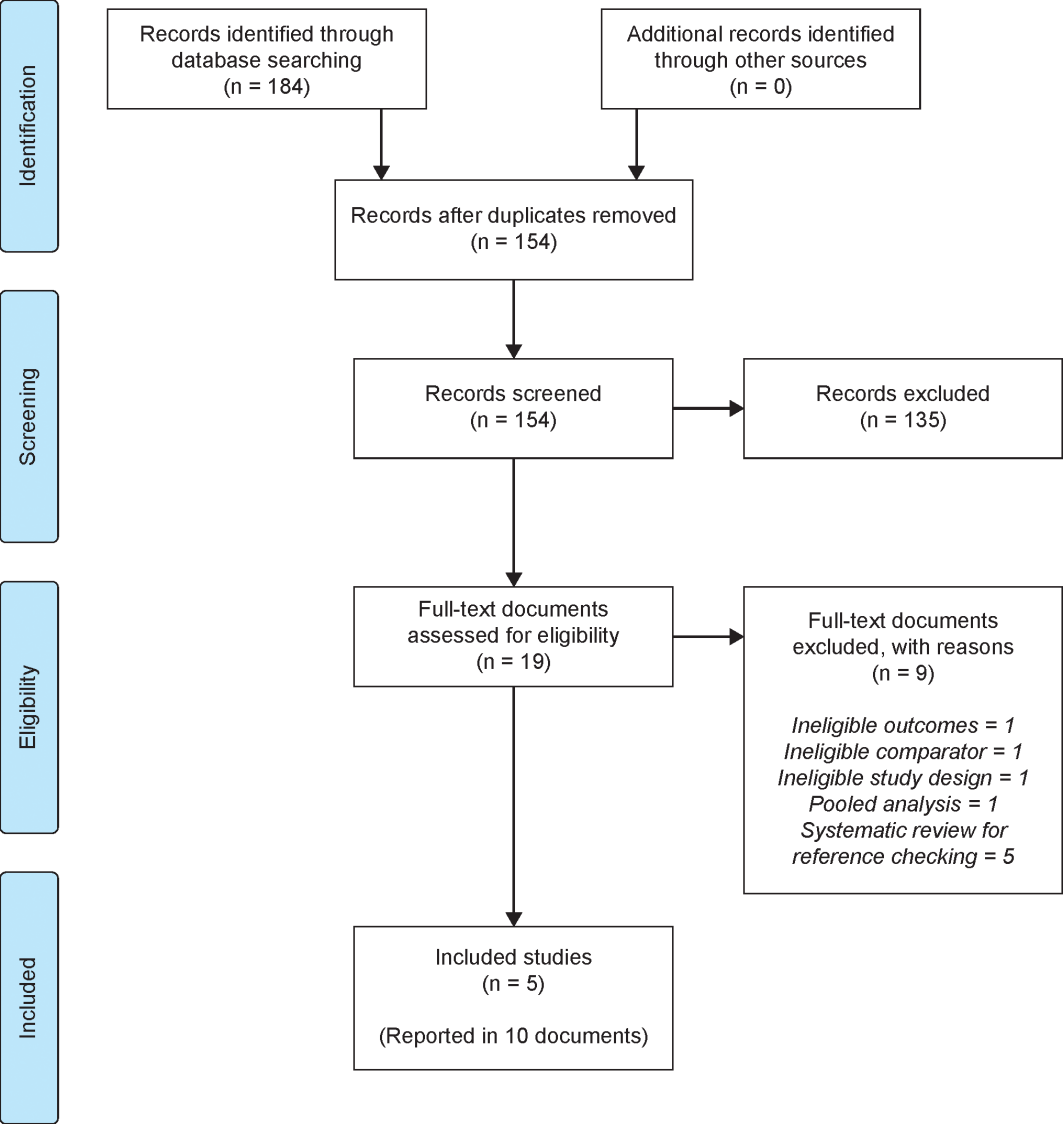
PRISMA Flow Diagram

Searches were conducted on November 20, 2018, with 95 records retrieved from MEDLINE and 89 records from CENTRAL. The literature search identified 154 unique records, including five studies that met eligibility criteria and were included in the analysis. Of these, two were tildrakizumab trials (reSURFACE 1 and reSURFACE 2)^13^ and three were guselkumab trials (VOYAGE 1,^16^ VOYAGE 2,^17^ and Ohtsuki et al.).^27^ The PRISMA study flow diagram (Figure 1) shows the number of records identified by the search and the numbers excluded at various selection stages (excluded studies are detailed in the Supplementary Material, Table S2). The trial reported by Ohtsuki and colleagues only included Japanese patients and was thus only considered in the sensitivity analysis.

The five trials were judged to have a moderate-to-low risk of bias (using the Cochrane Risk of Bias tool) based on factors including: having appropriate randomization, being double-blinded studies, having adequate concealment of treatment allocation, having care providers, participants and outcome assessors blinded to treatment allocation, having similar prognostic groups at study start, using ITT analysis, and having appropriate methods to account for missing data. Only two studies had unexpected imbalances in drop outs between groups (VOYAGE 1^16^ and Ohtsuki et al.).^27^ A summary of the risk of bias results is reported in the Supplementary Material (Table S3). The trials report outcomes at differing time points (Week 12 vs. 16, and 24 vs. 28) and using different dosing. Supplementary Table S4 shows the number of patients enrolled in each trial and their corresponding baseline characteristics. As stated, the population enrolled in the Ohtsuki study differed from that of the reSURFACE and VOYAGE trials as all patients in the Ohtsuki trial were Japanese. All other trials included patients of varying ethnicities. Weight, body mass index and disease duration were lower in the Ohtsuki study than the other trials.

### Bucher Indirect Comparison: Weeks 12–16

**Figure 2:**
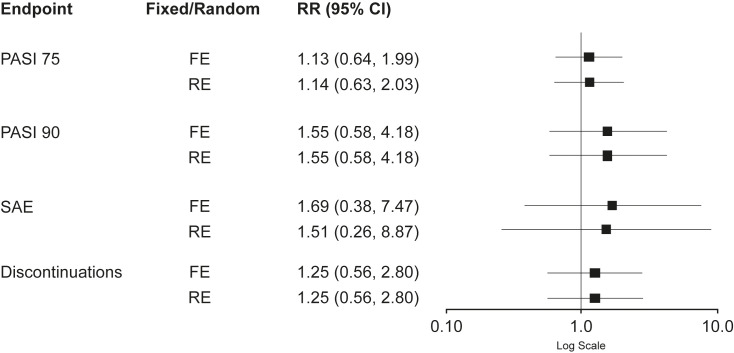
Comparisons of Endpoints: Guselkumab 100 mg vs. Tildrakizumab 100 mg (Weeks 12–16)^a^ Abbreviations: CI, confidence interval; FE, fixed effects; PASI, Psoriasis Area Sensitivity Index; PASI 75, ≥75% reduction in PASI; PASI 90, ≥90% reduction in PASI; RE, random effects; RR, risk ratio; SAE, serious adverse event. Values > 1 signify more events for guselkumab 100 mg. ^a^Endpoints examined were PASI 75, PASI 90, and frequency of SAE and discontinuations from pairwise meta-analyses analyses using either a FE or a RE model. Risk ratios are shown for each endpoint.

Forest plots presenting input study results and the I^2^ statistics demonstrated moderate heterogeneity for SAE tildrakizumab results (I^2^ 34.1%), indicating that both the FE and RE models could be considered. However, given the low heterogeneity in all other endpoints, the FE model could be considered for all endpoints including SAEs (Figure 2, Table 2). Comparisons with an I^2^ value of 0% indicated that the FE and RE models yielded identical results. Risk ratios for PASI 75 and PASI 90, using both models, were >1 and, therefore, favored guselkumab treatment. However, each of the 95% confidence intervals (CIs) contained 1, the risk ratio of identical risks; therefore, no significant difference could be detected between the two treatments for either PASI 75 (RR [95% CI]: FE 1.13 [0.64, 1.99]; RE 1.14 [0.63, 2.03]) or PASI 90 (RR [95% CI]: FE 1.55 [0.58, 4.18]; RE 1.55 [0.58, 4.18]) (Figure 2).

Risk ratios were also >1 for SAEs (RR [95% CI]: FE 1.69 [0.38, 7.47]; RE 1.51 [0.26, 8.87]) and treatment discontinuations (RR [95% CI]: FE 1.25 [0.56, 2.80]; RE 1.25 [0.56, 2.80]), indicating more events associated with guselkumab treatment in both models (favoring tildrakizumab). Again, no significant differences between treatments were detected (Figure 2). Calculation of ARRs for PASI 75, PASI 90, SAEs and treatment discontinuations demonstrated fewer events (as indicated by positive ARR values) for patients treated with tildrakizumab 100 mg versus guselkumab 100 mg. ARRs for FE and RE models respectively were: 10.2% and 10.8% for PASI 75; both 25.3% for PASI 90; 0.8% and 0.7% for SAEs; and both 0.6% for treatment discontinuations.

**Figure 3:**
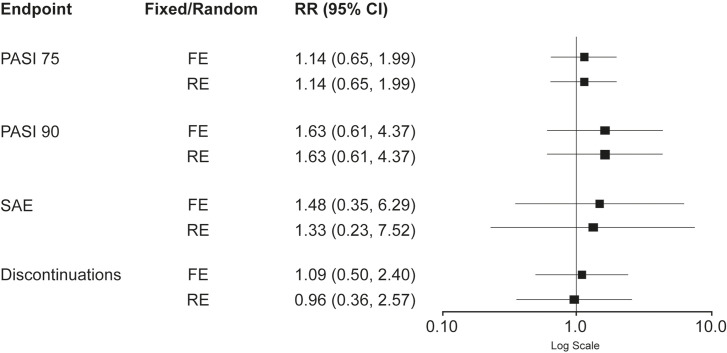
Comparison of Endpoints: Guselkumab 100 mg vs. Tildrakizumab 100 mg (Weeks 12–16) Sensitivity Analysis^a,b^ Abbreviations: CI, confidence interval; FE, fixed effects; PASI, Psoriasis Area Sensitivity Index; PASI 75, ≥75% reduction in PASI; PASI 90, ≥90% reduction in PASI; RE, random effects; RR, risk ratio; SAE, serious adverse event. Values >1 signify more events for guselkumab 100 mg. ^a^ The sensitivity analysis is based on the studies used for the “Weeks 12–16” analysis and the additional study (Ohtsuki et al. 2018) which included only Japanese patients and compared placebo and guselkumab 100 mg at Weeks 12–16. ^b^ Endpoints examined were PASI 75, PASI 90, and frequency of SAE and discontinuations from pairwise meta-analyses analyses using either a FE or a RE model. Risk ratios are shown for each endpoint.

Sensitivity analysis demonstrated that addition of the study by Ohtsuki and colleagues had little impact on heterogeneity apart from in treatment discontinuations (I^2^ 0% to 39.3%) and had no statistically significant impact on risk ratio results. However, the RE model for discontinuations showed more events with tildrakizumab (RR [95% CI]: 0.96 [0.36, 2.57]; ARR=–0.1%), but was not statistically significant (Table 2 and Figure 3).

### Bucher Indirect Comparison: Weeks 24–28

**Figure 4:**
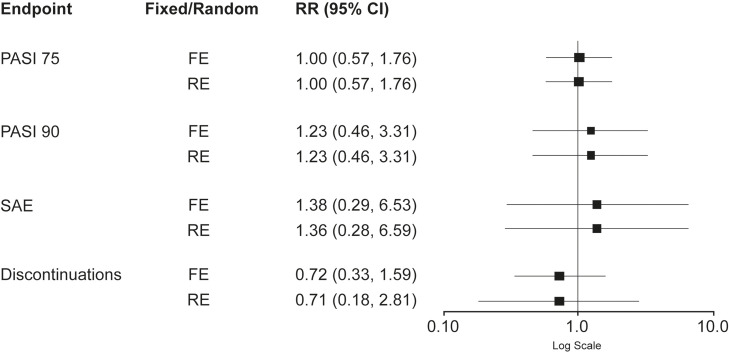
Comparisons of Endpoints: Guselkumab 100 mg vs. Tildrakizumab 100 mg (Weeks 24–28)^a^ Abbreviations: CI, confidence interval; FE, fixed effects; PASI, Psoriasis Area Sensitivity Index; PASI 75, ≥75% reduction in PASI; PASI 90, ≥90% reduction in PASI; RE, random effects; RR, risk ratio; SAE, serious adverse events. Values > 1 signify more events for guselkumab 100 mg. ^a^ Endpoints examined were PASI 75, PASI 90, and frequency of SAE and discontinuations from pairwise meta-analyses analyses using either a FE or a RE model. Risk ratios are shown for each endpoint.

There was a high amount of heterogeneity (I^2^) for discontinuations with tildrakizumab (84.5%) but not for other parameters (Table 2). Regarding PASI 75, risk ratios using both models were equal to 1 (95% CI: [FE: 0.57, 1.76; RE: 0.57, 1.76]), signifying the same number of events for both treatments with no significant difference detected. Risk ratios were >1 for PASI 90 (RR [95% CI]: FE 1.23 [0.46, 3.31]; RE 1.23 [0.46, 3.31]) and SAEs (RR [95% CI]: FE 1.38 [0.29, 6.53]; RE 1.36 [0.28, 6.59]) and there were more events associated with guselkumab treatment in both models. Again, no significant difference was detected. Considering treatment discontinuations, risk ratios were <1 (RR [95% CI]: FE 0.72 [0.33, 1.59]; RE 0.71 [0.18, 2.81]) and there were more events associated with tildrakizumab treatment in both models but, again, no significant difference was detected (Figure 4). Calculation of ARRs for PASI 75, PASI 90 and SAEs demonstrated the same number of events or fewer events for patients treated with tildrakizumab 100 mg versus guselkumab 100 mg. ARRs for FE and RE models respectively were: both 0% for PASI 75; both 14.4% for PASI 90; both 1.0% for SAEs. In contrast, the ARR for discontinuations demonstrated more events for patients treated with tildrakizumab 100 mg versus guselkumab 100 mg (FE, −1.1%; RE, −1.2%).

## DISCUSSION

This study describes the efficacy of guselkumab and tildrakizumab as per their pivotal clinical trials up to Week 28. Guselkumab trials (VOYAGE 1 and VOYAGE 2) reported data at Week 16 and Week 24, while tildrakizumab trials (reSURFACE 1 and reSURFACE 2) reported at Week 12 and Week 28. As such, Week 28 results are more representative of efficacy for tildrakizumab. Although the tildrakizumab trials evaluated 100 mg and 200 mg doses of tildrakizumab, we included only data for the 100 mg dose of tildrakizumab in our assessment, since this is the recommended dose in both the European Medicines Agency and the US Food and Drug Administration.^28^,^29^ Our study showed no statistically significant difference between guselkumab and tildrakizumab for PASI 75, PASI 90, SAE and rate of discontinuations at either 12 to 16 weeks or 24 to 28 weeks, timepoints that reflect those reported in the trials evaluated. Results from a network meta-analysis (NMA) that was conducted as part of a National Institute for Health and Care Excellence submission for tildrakizumab and an NMA of 77 psoriasis clinical trials, both of which included the trials analyzed in the present study among others, showed that tildrakizumab was inferior to guselkumab for all examined PASI levels.^30^,^31^ Furthermore, the analysis methods employed in the present study avoid some of the limitations regarding comparability of studies that may be associated with NMAs and, therefore, provide key additional data relating to the efficacy and safety of tildrakizumab that may assist decision-making processes. One limitation is the inclusion of Phase II studies; our analysis included only Phase III studies and response rates are often lower in Phase II trials than Phase III trials.^32^ Furthermore, only trials with licensed dosing regimens were included in the present study. Finally, the Ohtsuki trial that exclusively included Japanese patients was only included in the sensitivity analysis since different patient populations may exhibit dissimilar baseline characteristics and therefore respond differently to treatment.

The number of doses of guselkumab 100 mg and tildrakizumab 100 mg differed at the two timepoints. In the VOYAGE 1 and VOYAGE 2 trials, guselkumab 100 mg was given at baseline, Week 4 and subsequently every 8 weeks. As such, subjects had received three doses at Week 16 and five doses at Week 28. In the reSURFACE 1 and reSURFACE 2 trials, tildrakizumab 200 mg and 100 mg doses were given at baseline and Week 4 and subsequently every 12 weeks. As such subjects had received three doses at Week 16 and only four doses at Week 28 analysis, which may have influenced results. Greater improvement is usually seen at 28 weeks compared with 12 weeks of treatment.^30^

The limited number of publications available for inclusion in the current analysis impacts heterogeneity assessment and the relevance of the I^2^ statistic, since it is difficult to estimate variation between studies. Therefore, results for both the FE model and the RE model were reported. Interpretation of these results must therefore be considered in the context of a limited number of available studies. In addition, it is difficult to explore publication bias due to the small number of studies included here. The CIs for the results of the indirect comparison are very wide because the CIs for the individual risk ratios are very wide; this in turn is because the number of events in the placebo arms was always very low for each outcome. Thus, if the analysis had higher statistical power, significant differences between the two treatments may have been observed. Additionally, the amount of heterogeneity for discontinuations at Weeks 24 to 28 appears to be related to the low number of discontinuations for tildrakizumab in the reSURFACE 2 study. For this endpoint, the RE model should be considered. For all the other models the FE model was appropriate. Outcomes for placebo for Weeks 24 to 28 had to be imputed from data for Weeks 12 to 16 due to the discontinuation of the placebo arm. This imputation is consistent with the assumption that no changes are expected in the placebo arm beyond Weeks 12 to 16; however, this assumption cannot be verified.

FE and RE models yielded similar results apart from discontinuations in the sensitivity analysis. The study by Ohtsuki et al.^27^ met the eligibility criteria and was therefore included in this analysis. However, as the study is restricted to a Japanese population (and baseline characteristics are very different from the VOYAGE and reSURFACE trials) it may have limited relevance to tildrakizumab (Ilumetri^®^) and its European label.

We have described the efficacy of guselkumab and tildrakizumab up to 28 weeks. However, from a patient perspective it is important to evaluate long-term effects beyond half a year of treatment. Guselkumab and tildrakizumab have both demonstrated long-term (up to 3 years) efficacy in patients with psoriasis.^19^,^33^ The chronic and currently incurable nature of psoriasis is a long-term burden for patients who describe their worry about the future (including the decision to have children or not) and the destructive impact of psoriasis on multiple generations of their families.^34^ In conclusion, this study provides information regarding the efficacy and safety of tildrakizumab versus guselkumab in the treatment of moderate-to-severe psoriasis at two timepoints, Weeks 12 to 16 and Weeks 24 to 28. There was no statistically significant difference between guselkumab and tildrakizumab for PASI 75, PASI 90, SAE and rate of discontinuations at either timepoint. A head-to-head trial comparing guselkumab with tildrakizumab, including later timepoints, is warranted in future research. Direct and indirect comparisons of tildrakizumab and guselkumab with risankizumab may also be beneficial.

## Supplementary Information



## Figures and Tables

**Table 1:** Eligibility Criteria

	Inclusion Criteria	Exclusion Criteria
**Population**	Adult patients (≥18 years of age) with moderate-to-severe chronic plaque psoriasis	Children younger than 18 years
**Intervention**	Tildrakizumab (Ilumetri^®^) (100 mg Week 0, 4, Q12W) Guselkumab (Tremfya^®^) (100 mg Week 0, 4, Q8W)	Studies without either of these interventions
**Comparators**	Placebo Best supportive care^a^	NA
**Outcomes**	Severity of psoriasis: PASI 75 or 90 Frequency of Serious Adverse Events Withdrawals for any cause Outcomes based on ITT analysis	NA
**Study Design**	Phase III RCTs	Phase I, II, or IV RCTs Case studies Case reports Nonrandomized, controlled trials Reviews, including systematic reviews Observational studies
**Limits**	English language publications only Studies published as full text articles in peer review journals	Non-English language publications Conference abstracts

Abbreviations: ITT, intent-to-treat; NA, not applicable; PASI, Psoriasis Area and Severity Index; PASI 75, ≥75% reduction in PASI; PASI 90, ≥90% reduction in PASI; Q8W, dosed every 8 weeks; Q12W, dosed every 12 weeks; RCT, randomized, controlled trial.

a Best supportive care was not assessed in any studies as all were placebo-controlled trials.

**Table 2:** Heterogeneity Assessment and Sensitivity Analysis^a^ (Weeks 12–16 and Weeks 24–28)

Treatment 1	Treatment 2	I^2^ (Weeks 12–16)	I^2^ Sensitivity Analysis (Weeks 12–16)	I^2^ (Weeks 24–28)
**PASI 75**				
Placebo	Tildrakizumab 100 mg	0.0%	0.0%	0.0%
Placebo	Guselkumab 100 mg	9.3%	0.0%	0.0%
**PASI 90**				
Placebo	Tildrakizumab 100 mg	0.0%	0.0%	0.0%
Placebo	Guselkumab 100 mg	0.0%	0.0%	0.0%
**SAE**				
Placebo	Tildrakizumab 100 mg	34.1%	34.1%	3.9%
Placebo	Guselkumab 100 mg	0.0%	0.0%	NA
**Discontinuations**				
Placebo	Tildrakizumab 100 mg	0.0%	0.0%	84.5%
Placebo	Guselkumab 100 mg	0.0%	39.3%	NA

Abbreviations: NA, not applicable; PASI, Psoriasis Area and Severity Index; PASI 75, ≥75% reduction in PASI; PASI 90, ≥90% reduction in PASI; SAE, serious adverse event.

a The sensitivity analysis is based on the studies used for the “Weeks 12–16” analysis and the additional study (Ohtsuki et al. 2018) which included only Japanese patients and compared placebo and guselkumab 100 mg at Weeks 12–16.
