# Supplementary material for: A Systematic Literature Review and Bucher Indirect Comparison: Tildrakizumab versus Guselkumab
Source: J Health Econ Outcomes Res. 2020 Jul 24;7(2):123–9. doi: 10.36469/jheor.2020.13671 (PMC7398610; doi:10.36469/jheor.2020.13671)
Supplement: Supplementary file 1 [file jheor-7-2-13671-s01.pdf]

Journal of Health Economics and Outcomes Research

Supplementary Online Material

Du Jardin KG, Hurtado Lopez P, Lange M, McCool R, Maeso Naval S, Quickert S. A systematic literature review and Bucher indirect comparison: tildrakizumab versus guselkumab. *JHEOR*. 2020;7(2):123-129.
doi: 10.36469/jheor.2020.13671

Table S1. Details of Search Strategies
Table S2. Excluded Studies and Reasons for Exclusion
Table S3. Summary of Risk of Bias
Table S4. Participants' Baseline Characteristics

This supplementary material has been provided by the authors to give readers additional information about their work.

This is an open-access article distributed under the terms of the Creative Commons Attribution 4.0 International License (CCBY-4.0). View this license's legal deed at http://creativecommons.org/licenses/by/4.0 and legal code at http://creativecommons.org/licenses/by/4.0/legalcode for more information.

Table S1. Details of Search Strategies

Database	Search Strategy
Ovid MEDLINE(R) and Epub Ahead of Print, In-Process & Other Non-Indexed Citations and Daily <1946 to November 20, 2018>	#1 psoria$.ti,ab,kf. (43984)
	#2 exp Psoriasis/ (36568)
	#3 (Guselkumab or Tremfya$2 or CNTO 1959 or CNTO1959 or 089658A12D or 1350289-85-8).ti,ab,kf,nm,rn. (88)
	#4 (tildrakizumab or MK 3222 or MK3222 or SCH 900222 or SCH900222 or DEW6X41BEK or 1326244-10-3).ti,ab,kf,nm,rn. (59)
	#5 or/1-2 (49385)
	#6 or/3-4 (111)
	#7 5 and 6 (95)
Cochrane Central Register of Controlled Trials Issue 11 of 12, November 2018	#1 psoria* (6347)
	#2 [mh Psoriasis] (2797)
	#3 (Guselkumab or Tremfya* or "CNTO 1959" or CNTO1959 or 089658A12D or "1350289-85-8") (70)
	#4 (tildrakizumab or MK 3222 or MK3222 or SCH 900222 or SCH900222 or DEW6X41BEK or "1326244-10-3") (38)
	#5 #1 or #2 (6347)
	#6 #3 or #4 (100)
	#7 #5 and #6 in Trials (89)

Table S2. Excluded Studies and Reasons for Exclusion

Reference	Reason for Exclusion
Bilal J, Berlinberg A, Bhattacharjee S, Trost J, Riaz IB, Kurtzman DJB. A systematic review and meta-analysis of the efficacy and safety of the interleukin (IL)-12/23 and IL 17 inhibitors ustekinumab, secukinumab, ixekizumab, brodalumab, guselkumab and tildrakizumab for the treatment of moderate to severe plaque psoriasis. *J Dermatolog Treat*. 2018;29(6):569–78.	Systematic review for reference checking only
Blauvelt A, Reich K, Papp KA, et al. Safety of tildrakizumab for moderate-to-severe plaque psoriasis: pooled analysis of three randomized controlled trials. *Br J Dermatol*. 2018;179(3):615–22.	Pooled analysis
Hu C, Yao Z, Chen Y, et al. A comprehensive evaluation of exposure-response relationships in clinical trials: application to support guselkumab dose selection for patients with psoriasis. *J Pharmacokinet Pharmacodyn*. 2018;45(4):523–35.	Ineligible outcomes
Langley RG, Tsai TF, Flavin S, et al. Efficacy and safety of guselkumab in patients with psoriasis who have an inadequate response to ustekinumab: results of the randomized, double-blind, phase 3 NAVIGATE trial. *Br J Dermatol*. 2018;178(1):114–23.	Ineligible comparator
Nakamura M, Lee K, Jeon C, et al. Guselkumab for the Treatment of Psoriasis: A Review of Phase 3 Trials. *Dermatol Ther*. 2017;7(3):281–92.	Systematic review for reference checking only
Sofen H, Smith S, Matheson RT, et al. Guselkumab (an IL-23-specific mAb) demonstrates clinical and molecular response in patients with moderate-to-severe psoriasis. *J Allergy Clin Immunol*. 2014;133(4):1032–40.	Ineligible study design
Sbidian E, Chaimani A, Garcia-Doval I, et al. Systemic pharmacological treatments for chronic plaque psoriasis: a network meta-analysis. *Cochrane Database Syst Rev*. 2017;12:CD011535.	Systematic review for reference checking only
Tausend W, Downing C, Tyring S. Systematic review of interleukin-12, interleukin-17, and interleukin-23 pathway inhibitors for the treatment of moderate to severe chronic plaque psoriasis: ustekinumab, briakinumab, tildrakizumab, guselkumab, secukinumab, ixekizumab, and brodalumab. *J Cutan Med Surg*. 2014;18(3):156–69.	Systematic review for reference checking only

Table S3. Summary of Risk of Bias

**Trial Identifier**	**ReSURFACE 1**	**ReSURFACE 2**	**VOYAGE 1**	**VOYAGE 2**	**Ohtsuki 2018**
Was randomization carried out appropriately?	Yes	Yes	Yes	Yes	Yes
Blinding	Double blind	Double blind	Double blind	Double blind	Double blind
Was the concealment of treatment allocation adequate?	Yes	Yes	Yes	Yes	Yes
Were the groups similar at the outset of the study in terms of prognostic factors?	Yes	Yes	Yes	Yes	Yes
Were the care providers, participants and outcome assessors blind to treatment allocation?	Yes	Yes	Yes	Yes	Yes
Were there any unexpected imbalances in drop-outs between groups?	No	No	Yes	No	Yes
Is there any evidence to suggest that the authors measured more outcomes than they reported?	No	No	No	No	No
Did the analysis include an ITT analysis? If so, was this appropriate and were appropriate methods used to account for missing data?	Yes	Yes	Yes	Yes	Yes

Abbreviation: ITT, iIntent-to-treat.

Table S4. Participants' Baseline Characteristics

Trial identifier	Intervention	Number of patients randomised	Age Mean (SD) years	Female Number (%)	Ethnicity	Weight - kg Mean (SD)	BMI Mean (SD)	Disease duration Years Mean (SD)
ReSURFACE 1[13]	Tildrakizumab 200mg	308	46.9 (13.2)	82 (26.6)	White: 209 (68%)<br>Asian: 83 (27%)<br>Other: 16 (5%)	88·87 (24·09)	NR	NR
	Tildrakizumab 100mg	309	46·4 (13·1)	102 (33.0)	White: 217 (70%)<br>Asian: 70 (23%)<br>Other: 22 (7%)	88·53 (23·87)	NR	NR
	Placebo	155	47·9 (13·5)	55 (35.5)	White: 101 (65%)<br>Asian: 42 (27%)<br>Other: 12 (8%)	87·50 (26·04)	NR	NR
ReSURFACE 2[13]	Tildrakizumab 200mg	314	44·6 (13·6)	89 (28.3)	White: 284 (90%)<br>Asian: 14 (4%)<br>Other: 16 (5%)	88·35 (21·23)	NR	NR
	Tildrakizumab 100mg	307	44·6 (13·6)	87 (28.3)	White: 279 (91%)<br>Asian: 9 (3%)<br>Other: 19 (6%)	89·35 (22·12)	NR	NR
	Etanercept 50mg BIW for 12 weeks then QW	313	45·8 (14·0)	91 (29.1)	White: 289 (92%)<br>Asian: 10 (3%)<br>Other: 14 (4%)	87·97 (21·48)	NR	NR
	Placebo	156	46·4 (12·2)	44 (28.2)	White: 144 (92%)<br>Asian: 3 (2%)<br>Other: 9 (6%)	88·74 (22·73)	NR	NR
VOYAGE 1[16]	Guselkumab 100mg Q8W	329	43.9 (12.74)	89 (27.1)	Caucasian: 262 (79.6)<br>Black: 6 (1.8)<br>Asian: 51 (15.5)	NR	29.7 (6.22)	17.9 (12.27)
	Adalimumab 40mg Q2W	334	42.9 (12.58)	85 (25.4)	Caucasian: 277 (82.9)<br>Black: 8 (2.4)<br>Asian: 47 (14.1)	NR	29.8 (6.48)	17 (11.27)
	Placebo	174	44.9 (12.9)	55 (31.6)	Caucasian: 145 (83.3)<br>Black: 3 (1.7)<br>Asian: 23 (13.2)	NR	28.9 (6.89)	17.6 (12.44)
VOYAGE 2[17]	Placebo	248	43.3 (12.4)	75* (30.2)*	White: 206 (83.1)<br>Asian: 27 (10.9)<br>Black: 8 (3.2)	NR	29.6 (6.6)	17.9 (11.9)
	Guselkumab 100mg Q8W	496	43.7 (12.2)	147* (29.6)*	White: 408 (82.3)<br>Asian: 72 (14.5)<br>Black: 6 (1.2)	NR	29.6 (6.5)	17.9 (12.0)
	Adalimumab, 40mg Q2W	248	43.2 (11.9)	78* (31.5)*	White: 200 (80.6)<br>Asian: 37 (14.9)<br>Black: 5 (2.0)	NR	29.6 (6.6)	17.6 (11.7)
Ohtsuki 2018[27]	Placebo	64	48.3 (10.56)	10* (15.6)*	NR but narrative states all patients were Japanese	71.56 (14.01)	25.42 (4.791)	13.66 (10.291)
	Guselkumab 100mg Q8W	63	47.8 (11.07)	16* (25.4)*		74.27 (16.04)	26.33 (5.032)	14.39 (9.227)

Abbreviations: BIW, twice a week; BMI, body mass index; NR, not reported; PASI, Psoriasis Area and Severity Index; QW, once a week; Q2W, every 2 weeks; Q8W, every 8 weeks; SD: standard deviation.
